# Editorial: Drug resistance, global epidemiology and virulence of *Acinetobacter*

**DOI:** 10.3389/fmicb.2023.1151462

**Published:** 2023-02-21

**Authors:** Raffaele Zarrilli, Paolo Visca, Remy A. Bonnin, Emmanuelle Dé

**Affiliations:** ^1^Department of Public Health, University of Naples Federico II, Naples, Italy; ^2^Department of Sciences, Roma Tre University, Rome, Italy; ^3^Team “Resist” Unité Mixte de Recherche (UMR) 1184 “Immunology of Viral, Auto-Immune, Hematological and Bacterial Diseases (IMVA-HB),” Institut National de la Santé et de la Recherche Médicale (INSERM), Université Paris-Saclay, CEA, LabEx LERMIT, Faculty of Medicine, Le Kremlin-Bicêtre, France; ^4^University of Rouen Normandie, National Institute of Applied Sciences (INSA) Rouen Normandie, Centre National de la Recherche Science (CNRS), Lab. Polymers, Biopolymers, Surfaces (PBS), Unité Mixte de Recherche 6270, Rouen, France

**Keywords:** *Acinetobacter*, antimicrobial resistance, genomic epidemiology, virulence, environmental fitness

Bacteria belonging to the genus *Acinetobacter* are Gram-negative coccobacilli that are a frequent cause of health-care associated infections. *Acinetobacter baumannii* and the emergent species of *A. baumannii* (Ab) group like *A. pittii, A. nosocomialis, A. seifertii* and *A. lactucae* are the most clinically relevant species (Wong et al., 2017). Global epidemiology of *A. baumannii* showed a clonal population structure dominated by two major global clonal lineages (GC1 and GC2) and few additional epidemic clones (Gaiarsa et al., 2019). The most successful *A. baumannii* clones show resistance to a broad range of antimicrobials and disinfectants and share virulence features such as biofilm formation on abiotic surfaces, resistance to desiccation and adherence to epithelial cells, which contribute to their survival in the hospital environment and spread among patients (Giannouli et al., 2013).

This Research Topic collected original updates on the drug resistance, global epidemiology and virulence of *Acinetobacter*.

Three studies of This Topic investigated the genomics of antimicrobial resistance in *A. baumannii*. Vijayakumar et al. analyzed the mobile genetic elements associated with carbapenem resistance in *A. baumannii* clinical isolates from multiple hospitals in India between 2018 and 2019. They observed an increased prevalence of *bla*_*OXA*−23_ followed by dual carbapenemases, *bla*_*OXA*−23_, and *bla*_*NDM*_ and identified variations of AbaR4 and AbGRI resistance islands (RI). The majority of the isolates belonged to the dominant international clonal lineage 2, followed by less prevalent clones assigned to PasteurST25 and PasteurST10. Hamed et al. analyzed the genomic structure of RI in multidrug resistant and extensive drug resistant *A. baumannii* clinical isolates from Egypt. The majority of the isolates belonged to high-risk global clones (GC1, GC2, and GC9) and disclosed at least nine configurations of genomic RI, three of which (AbaR4, AbaR4b, and AbGRI1-like-2) carried *bla*_OXA − 23_ carbapenemase within Tn*2006*. An additional RI (RI-PER-7), carrying the resistance genes *armA* and *bla*_PER − 7_, was also identified on a plasmid into the strain M03. Yadav and Singh analyzed CRISPR-Cas type I-F1 and type I-F2 systems and its association with phage invasion in 4,977 genomes of *A. baumannii*. Of the 689 CRISPR-Cas positive genomes, 67.48% isolates harbored type I-F1, 28.59% had type I-F2, and 3.7% had co-existence of both type I-F1 and type I-F2 systems. A significantly reduced number of integrated phages in isolates with co-existence of type I-F1 + F2 compared with other counterparts was observed (*p* = 0.0041). In addition, the isolates carrying type I-F1 + F2 did not exhibit reduced resistance and virulence genes compared to CRISPR-Cas (–) and CRISPR-Cas (+) type I-F1 and type I-F2. This suggested that that the co-existence of CRISPR-Cas type I-F1 and F2 systems in *A. baumannii* imparts the hyperactivity against phages without affecting the presence of resistance genes.

The genetic elements responsible for horizontal gene transfer of antimicrobial resistance in *Acinetobacter* were also analyzed. Mindlin et al. showed that the population of natural *Acinetobacter* strains contains a significant number of conjugative mega-plasmids and revealed the presence of the genes for resistance to heavy metals in the plasmids from environmental strains, while the accumulation of antibiotic resistance genes carried by transposons and integrons in plasmids from clinical strains. Conjugative mega-plasmids may play a key role in the dissemination of multi-drug resistance among *Acinetobacter* species. The acquisition of blaOXA genes encoding different carbapenem-hydrolyzing class-D β-lactamases represents a main determinant of carbapenem resistance in *A. baumannii*. Giacone et al. investigated the contribution of pXerC/D-mediated recombination to the generation of structural diversity between resistance plasmids carrying pXerC/D-bounded blaOXA-58- and TnaphA6-containing resistance modules. They showed the existence of different pairs of recombinationally-active pXerC/D sites in these plasmids, some mediating reversible intramolecular inversions and others reversible plasmid fusions/resolutions.

Other studies investigated the role of efflux pumps (EPs) in antimicrobial resistance and tolerance to disinfectants in *A. baumannii*. The TetA(G) efflux pump of *A. baumannii* confers resistance to a variety of tetracyclines. Sumyk et al. studied the binding of tetracycline to TetR repressor of *A. baumannii* AYE (AbTetR). They showed that Arg104 and Arg135 residues, which are embedded at the entrance of the AbTetR binding pocket, play important roles in the recognition of tetracyclines, and act as a barrier to prevent the release of tetracycline from its binding pocket upon AbTetR activation. This might provide further insight for the development of new tetracycline antibiotics to overcome the efflux resistance mechanism deployed by *A. baumannii*. López-Siles et al. analyzed the promoter region markers associated with altered expression of three operons coding for Resistance-Nodulation-Division (RND) antibiotic efflux pumps (EPs) in *A. baumannii*. They *in silico* identified the genetic alterations leading to the constitutive upregulation of specific promoter regions of RND operons and then fused DNA of upstream sequences of RND operons to a luciferase reporter system. In sum, they developed a computational-experimental pipeline containing all components required for identifying the upstream regulatory resistome in *A. baumannii*. The management of infections caused by *A. baumannii* is hindered by its intrinsic tolerance to a wide variety of biocides. Migliaccio et al. investigated the role of different *A. baumannii* EPs in tolerance to chlorhexidine (CHX) and benzalkonium (BZK) and identified non-toxic compounds able to restore susceptibility to CHX and BZK in *A. baumannii*. They demonstrated that tolerance to CHX and BZK in *A. baumannii* ATCC 19606 was mediated by the activation of AdeB, AceI, and AmvA EPs, AdeB playing a major role. Importantly, inhibition of EP genes expression by either piperine or resveratrol at non-toxic concentrations restored CHX and BZK susceptibility in *A. baumannii*.

Several of the published manuscripts analyzed the molecular mechanisms of virulence in *A. baumannii*. To highlight critical molecular determinants of *A. baumannii* biofilm formation, Robin et al. used proteomic approaches on ATCC17978 and SDF strains. They identified the MacAB-TolC EP system as a contributor to biofilm formation on solid surfaces. Indeed, this EP is involved in the envelope stress response (maintenance of membrane rigidity, tolerance to high osmolarity conditions) but also in the maintenance of iron and sulfur homeostasis. This system could help *A. baumannii* to face deleterious conditions occurring in mature biofilms. Understanding regulation of genes involved in virulence and biofilm formation is essential to develop new strategies of infection control. It was previously shown that members of LysR-type transcriptional regulator (LTTR) family regulated numerous genes involved in these essential bacterial functions. To understand the genetic mechanisms regulating the interconvertion between virulent opaque (Vir-O) and avirulent translucent (AV-T) colony variants in *A. baumannii*, Tierney et al. examined the function of the LysR-type transcriptional regulator (LTTR), *ABUW_1132*. This global regulator, able to stimulate the expression of 74 genes by ≥2-fold, regulated positively the switch from Vir-O to AV-T and also impacted quorum sensing molecule secretion and surface-associated motility. Its deletion in AV-T variant promoted capsule formation and increased virulence. This suggests that AV-T variant, which has advantages in natural environments due to an increased ability to form biofilm, may also exist in virulent state, in case of *ABUW_1132* downregulation. LeuO, another LTTR, was characterized by Islam et al. *via* the construction of a *leuO* deletion mutant. Phenotypic characterization of this mutant showed that LeuO act as a repressor of biofilm synthesis by regulating genes within the *csuA/BABCDE* chaperon-usher pili system or the *A1S_0112-A1S_0119* acinetin operon, known as critical for biofilm formation. Several mutations in *leuO* gene from clinical strains were associated with a hyper-biofilm phenotype. Also, *leuO* gene disruption increased pathogenicity of *A. baumannii* in mouse infection model, while decreased motility and epithelial cell adhesion.

The molecular mechanisms responsible for environmental persistence were also investigated. Tajuelo et al. analyzed the role of the peptidoglycan recycling pathway enzymes AmpD and AnmK, which contribute to intrinsic fosfomycin resistance in *A. baumannii*, and also to virulence. They demonstrated that bacterial growth, fitness, biofilm formation and twitching motility were reduced in mutant strains *A. baumannii* ATCC 17978 Δ*ampD*::Kan and Δ*anmK*::Kan compared to the wild type strain. Also, Zhou et al. investigated the role of *gigA*/*gigB* genes of *A. baumannii* ATCC 17978, in bacterial growth, stress resistance, evading macrophage defense, and killing of *Galleria mellonella* larvae. The deletion of *gigA/gigB* conferred growth and replication defects within murine macrophages and an inability to kill *G. mellonella* larvae, while were dispensable for other stress-resistance survival phenotypes, including aminoglycoside resistance.

In conclusion, the spread of multi-drug resistant *A. baumannii* is a global public health threat. Understanding the mechanisms of antimicrobial resistance, virulence and adaptation to stressful conditions is important to prevent and control infections by this challenging pathogen.

## Author contributions

All authors listed have made a substantial, direct, and intellectual contribution to the work and approved it for publication.
